# Helios expression in naive CD4^+^ T cells decreases from neonates to older adults

**DOI:** 10.1093/cei/uxag041

**Published:** 2026-07-08

**Authors:** Hiroshi Mutoh, Takeo Mukai, Atsushi Ito, Tomoko Kawai, Hiromi Kamura, Kazuhiko Nakabayashi, Kohei Kashima, Mari Ichinose, Takayuki Iriyama, Shinya Takazawa, Mariko Yoshida, Yumi Tsuchida, Keishi Fujio, Naoto Takahashi

**Affiliations:** Department of Pediatrics, Faculty of Medicine, The University of Tokyo Hospital, Tokyo Japan; Department of Pediatrics, Faculty of Medicine, The University of Tokyo Hospital, Tokyo Japan; Department of Pediatrics, Faculty of Medicine, The University of Tokyo Hospital, Tokyo Japan; Department of Maternal-Fetal Biology, National Center for Child Health and Development, Tokyo Japan; Department of Maternal-Fetal Biology, National Center for Child Health and Development, Tokyo Japan; Department of Maternal-Fetal Biology, National Center for Child Health and Development, Tokyo Japan; Department of Pediatrics, International University of Health and Welfare Narita Hospital, Chiba Prefecture Japan; Department of Obstetrics and Gynecology, The University of Tokyo Hospital, Tokyo Japan; Department of Obstetrics and Gynecology, The University of Tokyo Hospital, Tokyo Japan; Department of Pediatric Surgery, The University of Tokyo Hospital, Tokyo Japan; Department of Pediatric Surgery, The University of Tokyo Hospital, Tokyo Japan; Department of Allergy and Rheumatology, The University of Tokyo Hospital, Tokyo Japan; Department of Allergy and Rheumatology, The University of Tokyo Hospital, Tokyo Japan; Department of Pediatrics, Faculty of Medicine, The University of Tokyo Hospital, Tokyo Japan

**Keywords:** Helios (IKZF2), naive CD4^+^ T cells, development, aging, immune tolerance

## Abstract

Fetuses, neonates, and infants are vulnerable to infectious diseases. At the same time, they are exposed to many harmless antigens; therefore, suppressing unnecessary immune responses to maintain immune tolerance is a rational strategy. In the elderly, chronic inflammatory and autoimmune diseases are more likely to occur due to inappropriate and dysregulated immune responses. Helios, encoded by the *IKZF2* gene, is a marker for natural regulatory T cells (Treg) but is also expressed at low levels in naive CD4^+^ T cells. Its expression decreases with age, which may contribute to an excessive immune response in older individuals. Flow cytometry and western blotting revealed that the decline in Helios expression begins in the early neonatal period and persists across subsequent life stages. Quantitative PCR showed that *IKZF2* expression was significantly and strongly negatively correlated with age (decreased by 40% per decade). Integration of DNA methylation array data and public datasets identified significant age-associated epigenetic modifications within the *IKZF2* gene body, which may partially explain the age-related reduction in its expression. Notably, these changes reflect a transition of naive CD4^+^ T cell characteristics: shifting from a “Treg-like” signature in neonates and infants—likely an adaptation to the extrauterine environment—toward a “memory-like” profile in older adults. In summary, Helios expression in human naive CD4^+^ T cells exhibits a continuous decline from the fetal stage through the neonatal, infant, childhood, adult, and elderly stages. This phenomenon may contribute to the shift from immune tolerance to immune response dominance over the course of aging.

## Teaser

Fetuses, neonates, and infants are vulnerable to infectious diseases. At the same time, they are exposed to many harmless antigens; therefore, suppressing unnecessary immune responses to maintain immune tolerance is a rational strategy. In the elderly, chronic inflammatory and autoimmune diseases are more likely to occur due to inappropriate and dysregulated immune responses. Helios, encoded by the IKZF2 gene, is a marker for natural regulatory T cells (Treg) but is also expressed at low levels in naïve CD4+ T cells. Its expression decreases with age, which may contribute to an excessive immune response in older individuals.

## Graphical Abstract

**Graphical Abstract:**
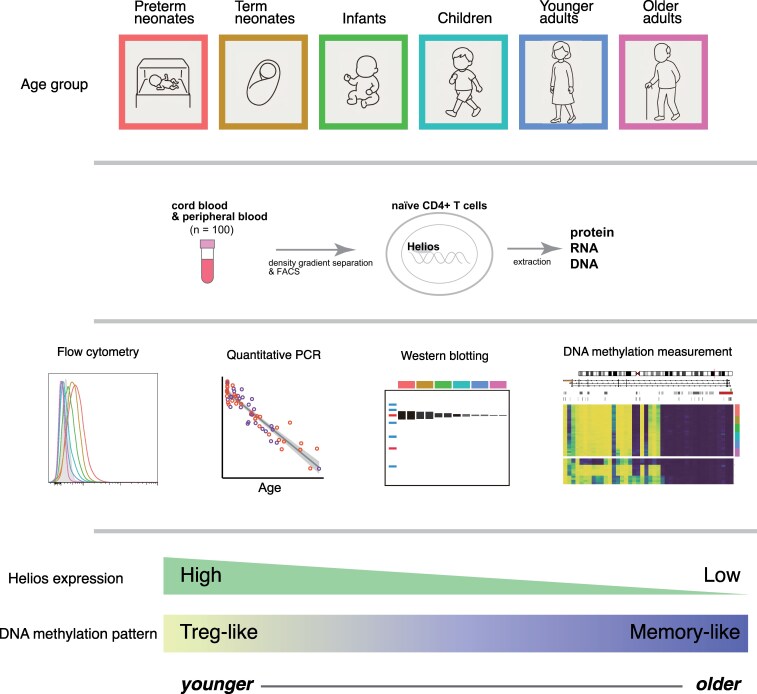

## Introduction

Some human immune functions change with growth and aging. The balance between immune tolerance and immune responses also changes [1].

During the fetal period, the fetus is protected *in utero* by the mother against infections from outside the body; however, even in normal pregnancy, the fetus is exposed to harmless maternal-origin antigens through the placenta [2]. This is often not a major clinical problem because of a high degree of immune tolerance. Pathogens, such as cytomegalovirus and *Toxoplasma gondii*, can invade deep into the fetus and cause congenital disorders. Severe cases of immune dysregulation, polyendocrinopathy, enteropathy, and X-linked syndrome can cause death *in utero* due to fetal edema [3, 4]. This indicates the importance of predominant immune tolerance during the fetal period.

Neonates and infants are exposed not only to pathogens but also to numerous innocuous commensal bacteria and food antigens [5–7]. Immune tolerance predominance is a reasonable mechanism to avoid unnecessary excessive immune responses to such antigens. However, neonates and infants often suffer from severe infections that are not seen in older children and younger adults [8–11].

After adolescence, when sufficient immune memory is stored and immune function has matured, immune tolerance is suppressed, and the immune response becomes stronger than that in younger age groups. This can prevent the invasion of infectious microorganisms, but adults can be more severely affected if they suffer from new infections with no immune memory. Examples include measles, rubella, mumps, varicella, and coronavirus disease 2019 [12–17]. In older age groups, because of inappropriate and uncontrollable hyperactivity of the immune response, not only infections but also chronic inflammation-related diseases and autoimmune diseases are more likely to occur. Thus, clinical evidence predicts a shift from immune tolerance dominance to immune response dominance from the fetal period to the neonatal, childhood, adulthood, and older ages. Until a certain point in time, these changes play a reasonable role in the external environment associated with growth.

A major role in immune tolerance is played by regulatory T cells. Helios, encoded by the Ikaros Family Zinc Finger 2 (*IKZF2*) gene, is a member of the Ikaros family of transcription factors. Helios is known as a marker for natural Tregs, and it has been reported that it contributes to the stability of forkhead box P3 (FOXP3), the master transcription factor of Tregs [18]. However, Helios is also slightly expressed in naive CD4^+^ T cells in adult humans and the decline in expression with aging is involved in the fate decision of inflammatory effector T cells [19]. The number of naive CD4^+^ T cells decreases with age due to thymic involution, and their production is compensated for by homeostatic proliferation; however, little is known about the changes from the neonatal period to adolescence [20].

*IKZF2* has been reported as one of many genes with higher expression in cord blood naive CD4^+^ T cells compared to adult peripheral blood, as demonstrated in some RNA-seq studies[21–23]. However, comparisons at the protein level (Helios) are limited [24], and clear age-related changes, particularly from neonates to childhood, have not been established.

We, therefore, aimed to evaluate Helios expression in naive CD4^+^ T cells in cord blood from preterm and term neonates and in peripheral blood from infants to older adults. We found that Helios expression was remarkably high during the neonatal period; however, it decreased significantly with growth and aging. We also confirmed that DNA methylation of *IKZF2* gene in naive CD4^+^ T cells undergoes similar age-related changes. Age-related changes in Helios expression in naive CD4^+^ T cells may be related to DNA methylation of the *IKZF2* gene, and the clinical response may contribute to the balance of immune tolerance and immune response.

## Materials and methods

### Participants

In this study, we collected and analyzed blood samples from 100 individuals who provided informed consent (Table 1). We divided the participants into six age groups based on the following definitions: preterm neonates, aged <36 weeks of gestational age; term neonates, aged ≥37 weeks of gestation; infants, aged 1 month to <2 years; children, aged 2–17 years; younger adults, aged 18–49 years; and older adults, aged ≥50 years. In some analyses, comparisons were made by dividing the participants into Groups 1 (preterm and term neonates), 2 (infants and children), and 3 (younger and older adults). There were limitations on the number of blood samples, particularly in preterm neonates, infants, and children, and on the number of samples that could be utilized for each test; therefore, not all tests could be performed on all samples.

**Table 1:** Sample information for each age group and study method.

		Group 1		Group 2		Group 3	
		Preterm neonates	Term neonates	Infants	Children	Younger adults	Older adults
Total	*n* (male/female)	16 (6/10)	14 (6/8)	18 (6/12)	19 (12/7)	24 (14/10)	9 (3/6)
	Age range	25–36 wk	37–41wk	2–20 mo	2–14 yr	18–49 yr	51–82 yr
FCM	*n* (male/female)	8 (3/5)	8 (3/5)	8 (2/6)	8 (5/3)	8 (5/3)	6 (2/4)
(Fig. 1)	Age range	25–34 wk	38–41wk	2–12 mo	6–11 yr	18–40 yr	51–66 yr
WB	*n* (male/female)	5 (1/4)	5 (2/3)	5 (3/2)	5 (4/1)	5 (3/2)	4 (1/3)
(Fig. 2b and c)	Age range	25–34 wk	38–40 wk	2–20 mo	2–13 yr	19 -32 yr	51 -66 yr
qPCR	*n* (male/female)	15 (6/9)	14 (6/8)	13 (4/9)	17 (10/7)	24 (14/10)	7 (/6)
(Fig. 2d and e)	Age range	25–36 wk	37–41 wk	2–20 mo	3–14 yr	18–49 yr	51–82 yr
DNAm	*n* (male/female)	7 (4/3)	5 (3/2)	5 (2/3)	5 (3/2)	5 (3/2)	5 (2/3)
(Figs 3 and 4)	Age range	31–36 wk	39–41 wk	2–20 mo	8–13 yr	22–32 yr	51–66 yr

FCM: flow cytometry, WB: western blotting, DNAm: DNA methylation array.

wk: weeks of gestational age, mo: months old, yr: years old.

### Ethics statement

We adhered to the Ethical Guidelines for Medical and Health Research Involving Human Subjects by the Ministry of Health, Labour, and Welfare, Japan, for all our methods. Our study was approved by the Human Genome Ethics Committee of The University of Tokyo Hospital (approval ID: 2022247NI) and Ethics Committee of National Center for Child Health and Development (approval ID: 2023-289).

We explained to the participants or their parents about blood collection and its use in our research and obtained their consent.

### Participants and study design

Cord blood was collected from the umbilical cords of newborns born at the University of Tokyo Hospital. Since almost all cases of preterm birth involve conditions that may affect the newborn's immune function, such as intrauterine infection or pregnancy-induced hypertension, which can cause preterm birth, or maternal treatments such as prenatal steroid administration, we also included such cases in our study. For term neonates, we excluded perinatal conditions that might have an impact on immune function and maternal medication (Supplementary Table S1). Peripheral blood samples of infants and children were collected in the Pediatric Department of the hospital. For infants and children, the effect of pain invasion was considered, and samples were collected at the same time as clinical blood sampling (Supplementary Table S2). Patients with suspected acute infectious or underlying diseases were excluded. For healthy adults, we recruited volunteers from the university and hospital staff. We excluded adults with acute or chronic diseases that affected their immune function.

### Mononuclear cell isolation and cryopreservation

We collected blood samples in EDTA-2K containing tubes. We isolated mononuclear cells using Lymphoprep (Serumwerk Bernburg AG) and SepMate (STEMCELL Technologies) and suspended in CELLBANKER® 1 plus (Nippon Zenyaku Kogyo). We preserved them in liquid nitrogen.

### FCM and cell sorting

The cryopreserved cells used for FCM were thawed and washed with PBS, and then incubated with Fc Receptor Binding Inhibitor Polyclonal Antibody (eBioscience) for 15 min. Viability staining with a Zombie Red™ Fixable Viability Kit (Biolegend) was performed using 1:100 dilution, and the cells were incubated at room temperature (20°C) for 15 min. Staining of surface markers was performed on ice for 20 min using the following anti-human monoclonal antibodies shown in Supplementary Table S3.

FCM sorting was performed using Moflo XDP (Beckman Coulter), and the data were analyzed using Summit software (Beckman Coulter) and FlowJo software V10 (BD Biosciences).

For intracellular staining, the Foxp3/Transcription Factor Staining Buffer Set (eBioscience) was used to fix and permeabilize the cell membrane after cell surface staining. Intracellular staining of the markers (Foxp3 and Helios) was performed on ice for 30 min (Supplementary Table S3, panel design 1). For the two intracellular staining assays, respective isotype controls were included (clones R35–95 from BD Biosciences and HTK888 from BioLegend) shown in Supplementary Figure S1.

Cells were sorted using FACS to evaluate Helios in the cell by methods other than FCM and intracellular staining and to evaluate *IKZF2* transcriptome and DNA modification. Naive CD4^+^ T cells were sorted as CD45^+^ CD3^+^ CD19^−^ CD4^+^ CD8^−^ CD25^−^ CCR7^+^ CD45RA^+^ (Supplementary Table S3, panel design 2). To sort the high-purity cells, stricter gates than the actual positive/negative boundaries we utilized, as shown in Fig. 2a.

For experiments requiring high cell yields, T cells were first enriched from mononuclear cells by immunomagnetic separation (EasySep™ Human T cell Isolation Kit; Stemcell Technologies). Subsequently, naive CD4^+^ T cells (CD3^+^ CD4^+^ CD8^−^ CD25^−^ CCR7^+^ CD45RA^+^) were isolated using FCM sorting as shown in Supplementary Figure S2a and Supplementary Table S3 (panel design 3).

### Protein extraction and western blotting

For protein extraction, cells were collected by FACS, the proteins were lysed in radioimmunoprecipitation assay buffer containing a protein inhibitor (Santa Cruz Biotechnology). Proteins were denatured in reducing conditions at 70°C for 10 min with 4× Bolt™ LDS Sample Buffer (Invitrogen) and 10× Bolt™ Sample Reducing Agent (Invitrogen).

Protein lysates equivalent to 2.4 × 10^4^ cells (or up to 2.0 × 10^5^ cells depending on the experiment) were subjected to SDS-PAGE using NuPAGE® Bis-Tris Precast Gel (Invitrogen) and subsequently transferred to polyvinylidene difluoride membrane using the iBlot 3 Gel Transfer Device (Invitrogen). For total protein normalization, membranes were stained with No-Stain Protein™ Labeling Reagent (Invitrogen) prior to blocking. The membrane was blocked in iBind™ Flex Solution, followed by overnight incubation with rabbit anti-human Helios (D8W4X) monoclonal antibody (Cell Signaling Technologies) and HRP-conjugated anti-rabbit IgG secondary antibody (Cell Signaling Technologies) using iBind Flex Western System (Invitrogen) and developed with chemiluminescence using the SuperSignal™ West Pico PLUS Chemiluminescent Substrate (Thermo Fisher Scientific). Chemiluminescent images were obtained using FUSION SOLO (Vilber Bio Imaging). After Helios detection, the membranes were stripped using the Restore Western Blot Stripping Buffer (Thermo Fisher Scientific). The same procedure was then used to detect β-actin using a rabbit anti-human β-Actin (13E5) monoclonal antibody (Cell Signaling Technologies) as the first antibody. In some cases, membranes were cut at the appropriate positions and incubated with a Histone H3 antibody (D1H2, Rabbit mAb; Cell Signaling Technology) along with Helios.

### RNA/DNA extraction and real-time qPCR

The sorted cells were collected directly into Buffer RLT Plus (Qiagen) and vortexed for lysis. Total RNA and DNA were prepared from cells per FACS-sorted sample using an Allprep DNA/RNA Micro Kit (Qiagen). cDNA synthesis was performed using the PrimeScript RT Reagent Kit (Takara Bio Inc.). mRNA transcript levels were determined via SYBR Green I technology using the SsoAdvanced Universal SYBR Green Supermix (Bio-Rad) on a CFX96 connected to a real-time PCR system (Bio-Rad), according to the manufacturer's protocol. The primer pairs (FASMAC) were shown in Supplementary Table S4.

*IKZF2* expression fold change was calculated via ΔCt analysis with *ACTB* as the housekeeping gene. The following equation was used to calculate the relative changes in gene expression:


ΔCt=(CtofIKZF2)−(CtofACTB)

$$IKZF2/ACTB\;=\;2^{\left(-\Delta \mathrm{Ct}\right)}$$

To further validate the robustness of our findings, additional reference genes, *RPL13A* and *TBP*, were utilized in sub-analyses.

### DNA methylation measurement

Genomic DNA was extracted, followed by bisulfite treatment using a Zymo EZ-96 DNA Methylation Kit (Zymo Research). The genome-wide DNA methylation status of >850 K CpG sites was analyzed using the Infinium MethylationEPIC BeadChip array (Illumina). Methylation data were acquired using the iScan system (Illumina) as idat files and processed using the ENmix package in R (v.4.3.2). The manifest file was annotated using “IlluminaHumanMethylationEPICanno.ilm10b4.hg19.”

Using preprocessENmix, we removed 15 235 probes because of their low quality. Additionally, we filtered 19 627 probes located on either the X or Y chromosome. Finally, 829 268 probes were used for further analysis. We used the beta value, which represents the ratio of methylated probe intensity to the overall intensity (sum of methylated and unmethylated probe intensities). The DNA methylation data are openly available in the NCBI GEO under accession number GSE288076.

### Statistical analysis

We conducted all statistical analyses in the R environment (v. 4.3.2). We used linear regression analysis or the Jonckheere–Terpstra test to determine trends in each age group. When comparing two groups, we used a *t*-test and, if necessary, made appropriate corrections using the Benjamini–Hochberg method.

## Results

### Flow cytometric evaluation demonstrates an age-associated decline in Helios protein expression within naive CD4^+^ T cells

We performed biological replicates (*n* = 6–8) for each age group (Table 1). The gating strategy is illustrated in Supplementary Figure S1a.

**Figure 1:**
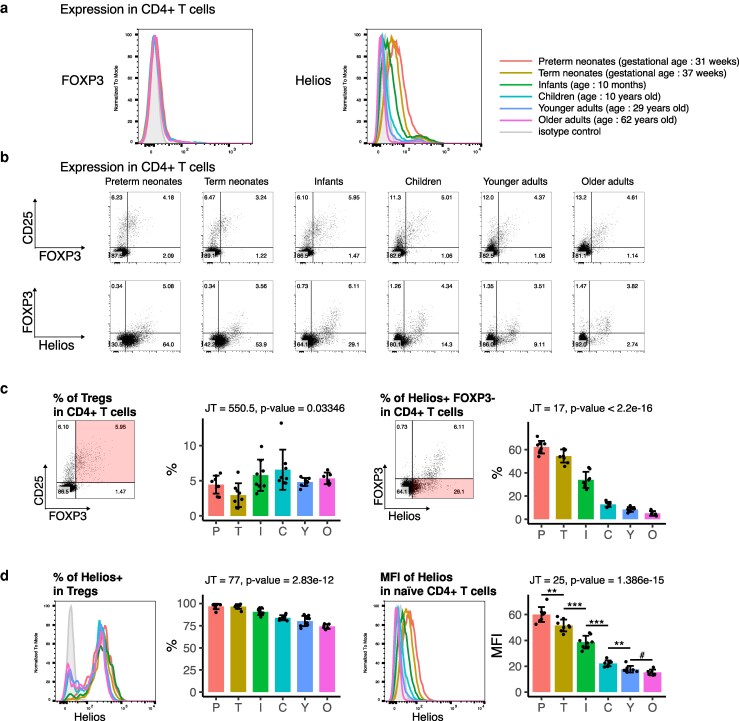
Younger age groups showed higher Helios expression in non-Tregs and naive CD4^+^ T cells, which decreased with age and growth. (a) FOXP3 and Helios expression in CD4^+^ T cells from each age group. (b) Representative flow cytometry plots of FOXP3, CD25, and Helios expression in CD4^+^ T cells from each age group. (c) Summary of expression described in (a). (d) Helios expression in Tregs, percentage of positive cells, Helios expression in naive CD4^+^ T cells, and mean fluorescence intensity (MFI). Comparisons between neighboring groups were made using the *t*-test (corrected using the Benjamini–Hochberg method). *: <0.05, **: <0.01, ***: <0.001, #: <0.1. P: preterm neonate; T: term neonate; I: infant; C: child; Y: younger adult; O: older adult; JT: Jonckheere–Terpstra test statistic. Bar plots: mean for each group, error bars: SD.

Representative examples of FOXP3 and Helios expression in the total CD4^+^ T cells from each age group are shown in Fig. 1a. The flow cytometric histograms of FOXP3 expression in CD4^+^ T cells exhibited a predominant negative peak that smoothly transitioned into a continuous positive fraction. Isotype controls from multiple age groups aligned with this negative population, and the negative peaks were nearly identical across all cases.

In contrast, Helios expression in CD4^+^ T cells exhibited a bimodal distribution characterized by two partially overlapping peaks, with the lower peak tending to be higher in the younger age groups (Fig. 1a). Tregs (FOXP3^+^ CD25^+^ CD4^+^ T cells) tended to decrease from preterm neonates to term neonates and then increase but generally made up approximately 5% of the cells in all age groups (Fig. 1b and c). When CD4^+^ T cells were analyzed for Helios and FOXP3, the proportion of Helios ^+^ FOXP3^−^ in CD4^+^ T cells, reflecting Helios expression in non-Tregs, was strongly related to growth and aging (Jonckheere–Terpstra test, Jonckheere–Terpstra statistic [JT] = 17, *P*-value <2.0 × 10^−16^).

Helios expression in Tregs was evaluated as the positive percentage (Fig. 1d). Approximately 75% of the adults were Helios-positive, as previously reported [25]. In contrast, almost 100% of neonates were Helios-positive, and the positive percentage decreased with growth and aging [24]. Helios expression in naive CD4^+^ T cells (CD45RA^+^ CCR7^+^ CD25^−^ CD4^+^ T cells) was evaluated using the mean fluorescence intensity. Younger age groups had higher Helios expression; the older age groups tended to have lower expression (JT = 25, *P*-value=1.4 × 10^−15^).

Collectively, these flow cytometric data demonstrate that Helios protein expression in naive CD4^+^ T cells progressively declines from the neonatal period through older adulthood.

### Immunoblotting validates a marked, lifespan-dependent reduction of Helios protein levels in purified naive CD4^+^ T cells

Helios in the cell lysates of sorted naive CD4^+^ T cells was quantified using the relative intensity between Helios and beta-actin. We utilized 29 samples for blotting: 15 samples were divided into two gels, and one sample (common sample) was evaluated on two gels, with four or five in each age group (Table 1). The sample on the other gel was adjusted based on the intensity ratio of the gels in a common sample.

**Figure 2:**
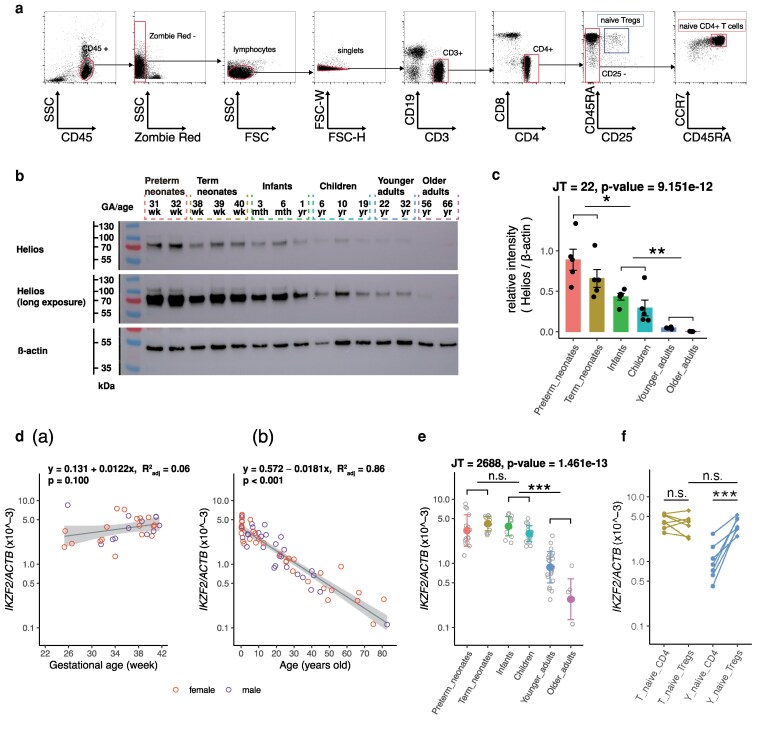
Helios and *IKZF2* gene expression in highly sorted naive CD4^+^ T cells was higher in younger age groups and decreased with growth and aging. (a) Sorting strategy for aimed cell subsets, naive CD4^+^ T cells, and naive Tregs. (b) Naive CD4^+^ T cells lysate from each age group were immunoblotted with anti-Helios Abs. Beta-actin was used as loading control. One of the two sets of data. (c) Summary of (b). Bar plots: mean for each group, error bars: SD. (d) The results of qPCR for cord blood and peripheral blood. *IKZF2*/*ACTB* was calculated as 10 ^ (−delta Cq), delta Cq = Cq of *IKZF2* − Cq of *ACTB*. (e) Summary of (d). (f) *IKZF2* expression of naive CD4^+^ T cells and naive Tregs derived from Term neonates and Younger adults. *: <0.05, **: <0.01, ***: <0.001, #: <0.1. JT: Jonckheere–Terpstra test statistic. Bar plots: mean for each group, error bars: SD.

The relative intensity of term neonates was 14 times higher than that of younger adults (mean 0.047) and approximately 200 times higher than that of older adults (mean 0.003), indicating a significant decrease in Helios expression with growth and aging (Fig. 2b and c).

In the comparison of the three groups (*t*-test, Benjamini–Hochberg method), there were significant differences between Groups 1 and 2 (*P* = 0.015) and between Groups 2 and 3 (*P* = 0.0024). The Jonckheere–Terpstra test (two-sided) showed a significant trend for growth and aging, with JT = 22 and *P* = 9.151 × 10^−12^. To validate our findings, we employed Histone H3 as an alternative internal control to beta-actin and performed total protein normalization using representative samples (*n* = 2 each for Groups 1, 2, and 3). As shown in Supplementary Figure S2a, these results confirmed a marked age-associated decrease, consistent with our initial observations.

Immunoblotting validated a marked, continuous reduction of Helios protein within naive CD4^+^ T cells, showing high consistency with the flow cytometric data.

### Quantitative PCR reveals a robust, continuous decadal decline in IKZF2 mRNA expression in postnatal naive CD4^+^ T cells

Transcriptional expression of *IKZF2* in naive CD4^+^ T cells was evaluated in 25 cord blood samples (Group 1) and 50 peripheral blood samples (Groups 2 and 3) using the ratio to the housekeeping gene *ACTB* (Table 1). In the evaluation of naive CD4^+^ T cells in Group 1, *IKZF2* expression showed no clear correlation with gestational age (linear regression analysis: adjusted *R*^2^ = 0.06, *P* = 0.100) (Fig. 2d(a)).

The variance of ΔCt (delta threshold cycle) for preterm neonates was 0.668, which was higher than 0.133 for term neonates. Conversely, in Groups 2 and 3 (infants and children, younger and older adults), there was an extraordinarily strong (*R*^2^ = 0.87) and significant (*P* < 0.001) correlation between age and *IKZF2*/*ACTB* (log scale). *IKZF2* expression decreased by 40% every 10 years (Fig. 2d(b)). This means that there was an approximately 8-fold difference in *IKZF2* expression between neonates and 40-year-old adults and a 60-fold difference between neonates and 80-year-old adults. However, there was a decrease in the mean value of *IKZF2*/*ACTB* with growth in the age group from term to childhood; however, this was not significant. There was also no significant difference in *IKZF2* expression between Groups 1 and 2 (Fig. 2e). No clear sex differences were observed in either cord or peripheral blood. To confirm our findings, we repeated the analysis using a combination of *RPL13A* and *TBP* as reference genes instead of *ACTB* (Supplementary Figure S2b). This alternative approach revealed age-associated changes that were highly consistent with the results obtained using *ACTB*.

**Figure 3:**
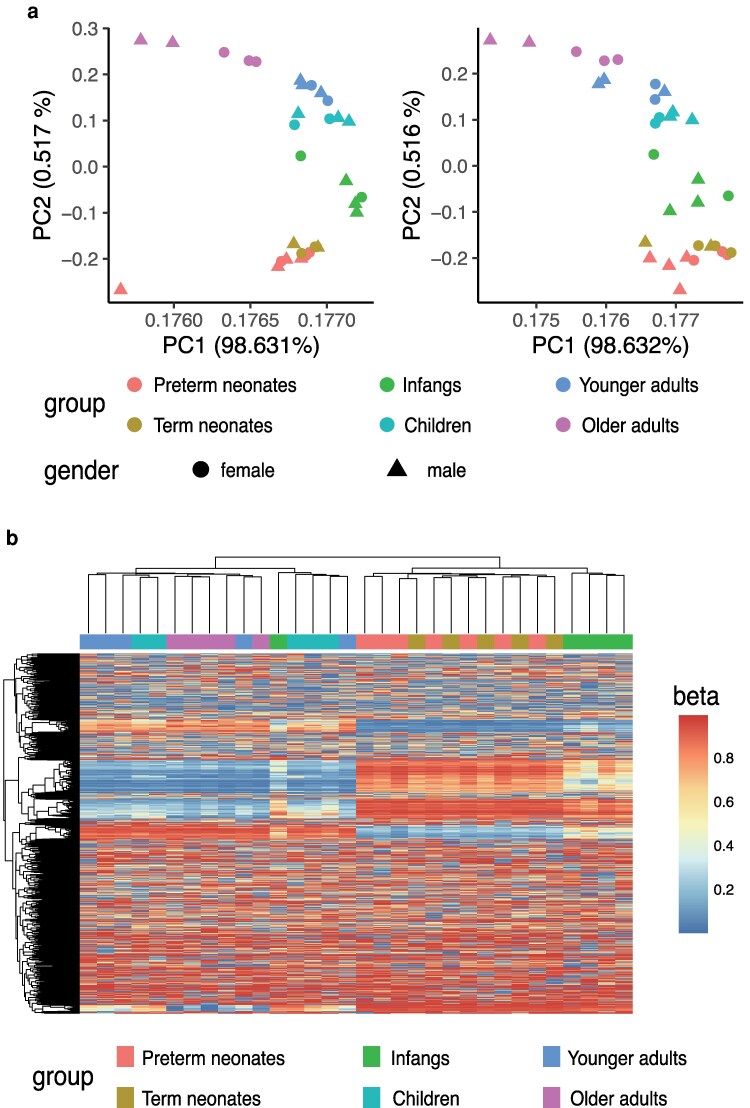
DNA methylation-based profiling revealed clusters for each generation group. (a) PCA without and with quantile normalization. (b) Heat map based on the top 2000 CpG sites with the highest variance in beta values.

Next, we compared *IKZF2* expression in naive CD4^+^ T cells and naive Tregs. As shown in Fig. 3f, *IKZF2* expression in naive CD4^+^ T cells was significantly lower (*P* = 0.0003) in younger adults, approximately one-eighth that in naive Tregs, as shown in previous reports [26, 27]. However, in term neonates, there was no significant difference in *IKZF2* expression between naive Tregs and naive CD4^+^ T cells (*P* = 0.40), although the FCM results (Fig. 1b) suggested that there was a difference in Helios expression between Tregs and non-Tregs (mostly naive CD4^+^ T cells in term neonates), even in term neonates. There was no significant difference in naive Tregs *IKZF2* expression between the two age groups.

In summary, these transcriptional analyses reveal a continuous, decadal decline in IKZF2 mRNA expression in naive CD4^+^ T cells after birth, while highlighting that neonates exhibit a uniquely high baseline expression level comparable to that of naive Tregs. In contrast to the protein-level findings from flow cytometry and western blotting, neonatal IKZF2 mRNA levels showed no significant correlation with gestational age.

### Comprehensive DNA methylation profiling delineates distinct lifespan-dependent epigenetic clusters in naive CD4^+^ T cells

Next, we performed a DNA methylation analysis to investigate its effects on *IKZF2* expression. As shown in Table 1, 32 samples were selected for naive CD4^+^ T cell methylation analysis, with 5–7 samples from each age group. There was no significant difference in the male-to-female ratio between groups. All samples were analyzed without any quality issues.

After filtering, PCA was performed using all cytosine-phosphate-guanine (CpG) sites (Fig. 3a). The PCA results are shown for two cases: one with quantile normalization and the other without. In both cases, the first principal component (PC1, x-axis), which is over 98%, shows a difference between older adults and the other groups and is thought to show the effects of aging. This trend was more clearly observed in the case without quantile normalization. Conversely, the second principal component (PC2, y-axis) was ordered from the youngest preterm newborn to the oldest adult in both PCAs. This trend was observed for both growth and aging.

In the heatmap in Fig. 3b, we analyzed using the top 2 K of the variance in beta values at 850 K. Similar to the PCA results, clusters were formed within the same or adjacent age groups.

Taken together, these global epigenetic data indicate that human naive CD4^+^ T cells undergo systemic DNA methylation remodeling during growth and aging, forming distinct epigenetic clusters that precisely reflect developmental and chronological stages.

### Targeted epigenetic mapping of the IKZF2 locus reveals an age-dependent shift from a Treg-like to a memory-like methylation profile within the gene body

**Figure 4:**
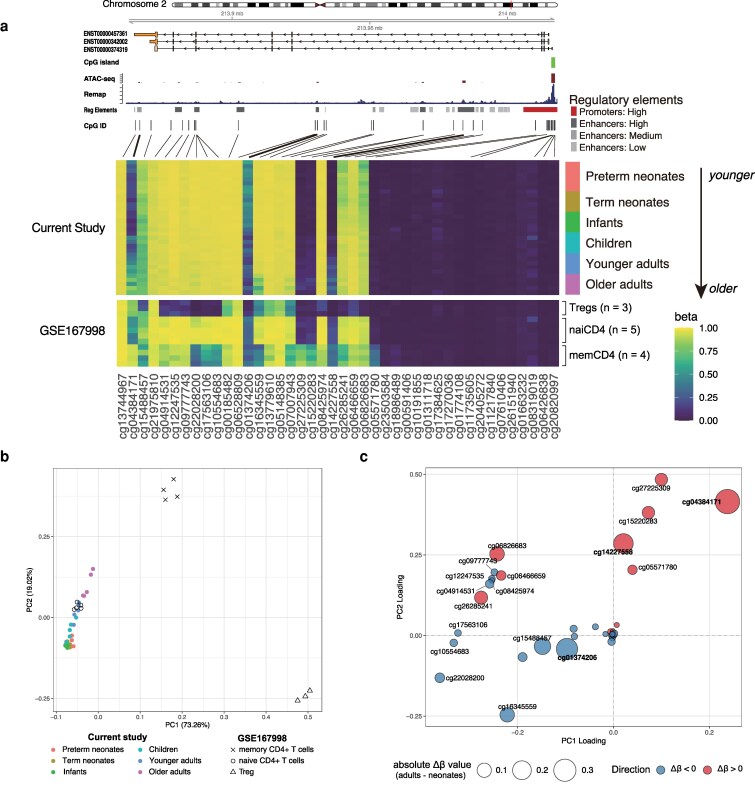
DNA methylation of the *IKZF2* gene changed with growth and aging. Methylation changes were observed in the open chromatin region of the gene body. (a) Gene map and heatmap of the methylation profile of the *IKZF2* gene. CpG island: chr2:214016358-214017105; ATAC-seq: data from GSE126113, average peak values from two healthy adults; ReMap: a density plot of transcription factors; Regulatory elements: confidence scores for GeneHancer promoter and enhancer regulatory elements are color-coded as shown on the right. CpG ID: 43 CpG sites available for analysis in this study. The CpG sites are arranged in order on the chromosome. The groups are arranged from younger to older, and within each group, they are arranged from younger to older. The two heatmaps represent the beta values for the current study (*n* = 32; all naive CD4^+^ T cells) and the reference dataset (*n* = 10; Tregs, naive CD4^+^ T cells, and memory CD4^+^ T cells). Values are shown without normalization and are color-coded according to the legend on the right. Clustering was not performed on either of the axes. (b) PCA of samples from the current study (*n* = 32) and reference dataset (*n* = 10), showing clustering by cell type and age group. (c) Bubble chart representing PC1 and PC2 loadings for individual CpG sites from the PCA in (b). The size and color of each bubble indicate the absolute value and direction (sign) of Δβ, respectively, as shown in the volcano plot (Supplementary Figure S3b).

The methylation profile of the *IKZF2* gene locating in chromosome 2 (chr2:213 864 423–214 017 180; hg19) is shown in Fig. 4. Among the 44 CpG sites identified within this region, one site (cg02239891) was excluded due to low technical quality, leaving 43 sites for subsequent analysis. The methylation levels (beta values) for these sites are presented in the heatmap in Fig. 4a, with corresponding *z*-scores shown in Supplementary Figure S3a.

To evaluate whether age-associated CpG sites correlate with *IKZF2* transcriptional regulation, we integrated three functional genomic datasets into our analysis. Open chromatin regions in naive CD4^+^ T cells were identified using publicly available ATAC-seq data (GSE126113) [28], representing the mean peak values from two healthy adults. Transcription factor density was further assessed using ReMap 2022 [29], an integrative atlas of ChIP-seq datasets, while *IKZF2* regulatory elements and their associated confidence scores were identified using GeneHancer data obtained via the UCSC Genome Browser [30].

To further explore the relationship between age-related methylation changes and T-cell differentiation, we compared our data with an Infinium MethylationEPIC BeadChip array dataset (GSE167998) comprising sorted CD4^+^ T-cell subsets from healthy adults[31]. These reference subsets included regulatory T cells (CD25^+^ CD127^−^ CD4 ^+^T cells), naive CD4^+^ T cells (CD45RA ^+^ CD4^+^ T cells), and memory CD4^+^ T cells (CD45RA^−^ CD4^+^ T cells). As shown in the heatmap (Fig. 4a) and principal component analysis (PCA) (Fig. 4b), we observed a strong concordance in the methylation patterns of naive CD4^+^ T cells between our study and the reference dataset. Additionally, to contextualize these findings within global methylation changes, we compared beta values of 820 K CpG sites between adults (young and elderly) and neonates (preterm and term). The resulting volcano plot highlighted significant differential methylation (Δβ), with sites specifically related to *IKZF2* being prominently represented (Supplementary Figure S3b). Notably, the *IKZF2* promoter region (chr2:214 015 971–214 017 336), which encompasses a CpG island (light green in Fig. 4a), was found to be predominantly demethylated across all three cell types in both our samples and the reference data. Furthermore, as shown in Supplementary Figure S3a, comparisons of *z*-scores confirmed the absence of significant age-related differences within the promoter region.

The majority of age-associated CpG sites identified in this study were also differentially methylated among the three T-cell subsets in the reference data. These sites were primarily localized within the gene body rather than the promoter region. PCA revealed that our samples clustered by age group, with the methylation profile of naive CD4^+^ T cells shifting along the PC2 axis from negative to positive values as individuals progressed from neonate to old age (Fig. 4b). This shift indicates a transition from a pattern resembling regulatory T cells (Tregs) toward one more characteristic of memory CD4^+^ T cells. Quantitative analysis of Euclidean distances further supported this transition, showing that the *IKZF2* methylation profile moves away from a “Treg-like” state and toward a “memory-like” state with advancing age (Supplementary Figure). Analysis of the contribution of individual CpG sites to these shifts showed that those with the highest PC2 loadings often exhibited relatively large age-dependent changes (Δβ), as shown in Fig. 4c. Although some exceptions such as cg05571780 and cg22028200 were observed, most representative sites with high contribution (e.g. cg27225309, cg04384171, cg15220283, cg14227558, and cg06826683) were situated within open chromatin regions of naive CD4^+^ T cells, transcription factor binding sites, or high-to-moderate score GeneHancer regulatory elements. Notably, the sign of Δβ was clearly segregated according to the PC2 polarity; the majority of CpG sites with positive PC2 loadings exhibited positive Δβ, whereas those with negative loadings showed negative Δβ. Collectively, these findings indicate that the DNA methylation pattern of the *IKZF2* locus in younger individuals is more similar to that of Tregs than memory CD4^+^ T cells. While the age-related changes occur predominantly in the gene body rather than the promoter, these shifts involve key regulatory regions, suggesting their potential involvement in modulating *IKZF2* expression across the lifespan.

Collectively, these targeted epigenetic findings indicate that the DNA methylation pattern of the IKZF2 locus in younger individuals sustains a “Treg-like” signature within key open-chromatin gene body regions, which progressively transitions toward a “memory-like” profile during aging, potentially driving the long-term modulation of IKZF2 expression across the lifespan.

## Discussion

Naive T cells comprise most T cells in newborns; however, the proportion and absolute number of naive T cells in peripheral blood decrease due to thymus involution with aging [32]. After adulthood, naive T cells are maintained by antigen-independent homeostatic proliferation in the periphery, which can lead to a decrease in the TCR repertoire and immunocompromization [20, 33]. CD4^+^ T cells from the cord and fetal blood tend to be more easily induced to differentiate into Tregs *in vitro* than CD4^+^ T cells from adult peripheral blood [34, 35]. There are also reports that Treg-related genes are more highly expressed in younger people [21]. Various factors can cause chronic inflammatory reactions with aging, and qualitative changes in naive T cells may be one of them [36].

Mutations in *IKZF2* (Helios) have been linked to immune disorders in a few cases [37, 38], although the clinical manifestations associated with Helios are notably rarer than those of other IKZF family members, such as Ikaros and Aiolos [39–41]. Helios-knockout (KO) mice have a high mortality rate during the neonatal period [42]. In contrast, Treg-specific Helios KO mice do not show any significant autoimmune symptoms during childhood; however, various immune abnormalities are observed with aging [43]. These results suggest that Helios is important in non-Treg cells during the fetal and neonatal periods. Recent studies have reported Helios expression in the naive T cells of young adults and its association with aging, and it is possible that it is involved in age-related changes in immune responses [19, 25]. Our results extend these findings, demonstrating that Helios expression in naive CD4^+^ T cells decreases not only during adulthood but also progressively during the transitions from neonate to child and child to adult. The observed difference between preterm and full-term neonates further suggests that Helios downregulation may begin during fetal development. This transition—from a state of immune tolerance dominance toward immune response dominance—likely represents an adaptive functional maturation rather than a simple cellular malfunction.

Although the present study did not directly investigate the functional roles of Helios within naive CD4^+^ T cells, its age-associated decline likely reflects a direct functional loss or a broader epigenetic transition. First, although previous work on adult aging demonstrated that Helios regulates naive CD4^+^ T cell fate and maintains cellular quiescence by modulating IL-2R–STAT5 signaling and chromatin remodeling [24], whether similar mechanisms operate from neonates to childhood has remained unknown and warrants further functional validation. Second, rather than directly driving this process, the decline in Helios may instead reflect a global shift of the naive T cell landscape from a Treg-like toward an effector-like state.

In this study, Helios, protein level, as evaluated by flow cytometry and western blotting, were highest in preterm neonates and decreased significantly across the lifespan. Interestingly, *IKZF2* mRNA levels did not show a significant difference between preterm and term neonates, and the decreasing trend in infants and children did not reach statistical significance. This discrepancy suggests that post-translational mechanisms or rapid transcriptional changes may play a more dominant role in the early neonatal period.

To investigate the epigenetic basis of these changes, we focused on DNA methylation, a stable yet dynamic modification typically associated with gene silencing at promoters and transcriptional elongation in gene bodies [44–47]. While many aging studies utilize whole blood or mononuclear cells—which are susceptible to age-related shifts in cell composition—we analyzed highly purified naive CD4^+^ T cells to isolate cell-specific epigenetic trends. Aging was associated with increased methylation entropy and hypomethylation in highly methylated regions as in previous reports [48–51]. However, we also identified specific CpG sites that undergo significant methylation changes beginning in early childhood.

This epigenetic remodeling extended to IKZF2-related regions. We demonstrated that age-related methylation shifts in naive CD4^+^ T cells involve a transition from a “Treg-like” (IKZF2-positive) toward a “memory-like” (IKZF2-negative) profile. Specifically, the age-associated increase in gene body methylation correlated with a shift toward patterns characteristic of memory CD4^+^ T cells. Notably, we observed a clear segregation in the sign of Δβ according to PC2 polarity: sites with positive PC2 loadings (favoring a memory-like state) generally exhibited positive Δβ, while those with negative loadings showed negative Δβ. These results suggest that in younger individuals, naive CD4^+^ T cells maintain an epigenetic environment that sustains a Treg-like signature through specific gene body methylation patterns.

Although gene body methylation generally correlates with transcriptional activity [46, 47], it may modulate IKZF2 expression through complex mechanisms such as altering three-dimensional chromatin architecture or transcription factor recruitment [52]. Interestingly, the IKZF2 methylation patterns in neonates and infants were nearly indistinguishable. While DNA methylation likely accounts for the gradual, decadal decline of IKZF2 expression in adults, it does not fully explain the rapid downregulation of Helios protein observed between the neonatal and infant periods. This implies that transient transcriptional or post-translational regulators may dominate Helios expression in early life, with DNA methylation serving as a stabilizing mechanism for long-term silencing in later years.

The consistent decrease in Helios expression from preterm neonates to older adults suggests a shift from immune tolerance to immune response dominance [19]. This transition is vital for host defense but may also contribute to chronic inflammation, which impacts systemic aging beyond the immune system.

Several limitations of this study should be noted. The small volume of blood obtainable from preterm infants and children limited the scope of our examinations. Furthermore, our cross-sectional design means we did not track individual longitudinal changes. While we focused on “healthy” subjects, clinical assessments of “non-acute” status have inherent limitations. Finally, the methylation array covers only 3–5% of total CpG sites and is biased toward promoter regions, potentially omitting key regulatory elements in other genomic areas.

## Supplementary Material

uxag041_Supplementary_Data

## Data Availability

The DNA methylation data generated during this study have been deposited in the NCBI Gene Expression Omnibus (GEO) and are openly available under accession number GSE288076.

## Abbreviations

CpG: cytosine-phosphate-guanine
DNAm: DNA methylation array
FCM: flow cytometry
FOXP3: forkhead box P3
IKZF2: Ikaros Family Zinc Finger 2
JT: Jonckheere–Terpstra test statistic
KO: knockout
MFI: mean fluorescence intensity
mo: months old
PC1: first principal component
PC2: second principal component
PCA: principal component analysis
SD: standard deviation
Treg: regulatory T cell
WB: western blotting
wk: weeks of gestational age
yr: years old
ΔCt: delta threshold cycle
