# Supplementary material for: Helios expression in naive CD4+ T cells decreases from neonates to older adults
Source: Clin Exp Immunol. 2026 Jul 8;220(1):uxag041. doi: 10.1093/cei/uxag041 (PMC13415459; doi:10.1093/cei/uxag041)
Supplement: uxag041_Supplementary_Data [file uxag041_supplementary_data.zip › Supplementary Table.docx]

Supplementary Table 1. Background of Group 1 (preterm and term neonates)

|  | Preterm neonates | Term neonates |
| --- | --- | --- |
| Total numbers, n | 16 | 14 |
| Gestational age, max - min | 25w2d – 36w2d | 37w5d – 41w4d |
| Birth weight (g) | 441 – 2644 | 2686 – 4167 |
| Low birth weight infants, n | 15 | 1 |
| Very low birth weight infants, n | 7 | 0 |
| Extremely low birth weight infants, n | 4 | 0 |
|  |  |  |
| Types of delivery |  |  |
| Vaginal delivery, n | 1 | 6 |
| Elective Caesarean section, n | 2 | 2 |
| Emergency Caesarean section, n | 12 | 6 |
|  |  |  |
| Umbilical cord arterial pH (min - max) | 7.20 – 7.36 | 7.09 – 7.37 |
| Apgar score at 1 min, min - max | 5 - 8 | 1 - 9 |
| Apgar score at 5 min, min – max | 7 - 9 | 8 - 9 |
|  |  |  |
| Maternal complications and therapies, n |  |  |
| Hypertensive disorders of pregnancy | 4 | 0 |
| Pathological chorioamnionitis* | 2 | 1 |
| Maternal auto-immune disease | 1 | 0 |
| Antenatal corticosteroid therapy | 9 | 0 |
|  |  |  |
| Admission to neonatal intensive care unit, n | 14 | 1 |

w: weeks, d: days

* Placental pathological examinations were mandatory for all preterm infants born at 35 weeks or earlier. In contrast, examinations for those born at 36 weeks or later were performed solely based on the clinician's judgment of necessity.

Supplementary Table 2. Background of Group 2 (infants and children)

|  | Infants | Children |
| --- | --- | --- |
| Total numbers, n | 18 | 19 |
| Age, max - min | 2 – 20 months | 2 – 14 months |
|  |  |  |
| Blood sampling time, n |  |  |
| Antibody test (negative results) |  |  |
| Congenital toxoplasma infection | 12 | 0 |
| Congenital syphilis | 3 | 0 |
| Pre-operation |  |  |
| Cryptorchidism | 2* | 1 |
| Inguinal hernia | 2* | 4 |
| Hydrocele | 0 | 2 |
| Ophthalmological diseases | 0 | 2 |
| Congenital nevus | 0 | ３ |
| Orthopedic diseases | 0 | 3 |
| Congenital microtia | 0 | 3 |
| Pre-examination |  |  |
| Retrograde urethrography | 0 | 1 |

*One case was combined of cryptorchidism and inguinal hernia.

**Supplementary Table 3.**

Flow cytometry antigens panel design 1.

| Antigen | Antigen Localization | Fluorescent dye | Antibody clone | Source |
| --- | --- | --- | --- | --- |
| CD3 | Cell surface | FITC | UCHT1 | Biolegend |
| CD45RA | Cell surface | PerCP | HI100 | Biolegend |
| CD4 | Cell surface | APC/Fire 750 | RPA-T4 | Biolegend |
| CD25 | Cell surface | Brilliant Violet 421 | BC96 | Biolegend |
| CCR7 | Cell surface | Brilliant Violet 510 | G043H7 | Biolegend |
| FOXP3 | Nucleus | PE | PCH101 | eBioscience |
| Helios | Nucleus | APC | 22F6 | Biolegend |

Flow cytometry antigens panel design 2 (Cell sorting).

| Antigen | Antigen Localization | Fluorescent dye | Antibody clone | Source |
| --- | --- | --- | --- | --- |
| CCR7 | Cell membrane | FITC | G043H7 | Biolegend |
| CD25 | Cell surface | PE | BC96 | Biolegend |
| CD3 | Cell surface | PE/Cyanine7 | UCHT1 | Biolegend |
| CD19 | Cell surface | PerCP-Cyanine5.5 | HIB19 | Biolegend |
| CD45 | Cell surface | APC | HI30 | Biolegend |
| CD4 | Cell surface | APC/Fire750 | RPA-T4 | Biolegend |
| CD45RA | Cell surface | Brilliant Violet 421 | HI100 | Biolegend |
| CD8a | Cell surface | Brilliant Violet 510 | RPA-T8 | Biolegend |

Flow cytometry antigens panel design 3 (Cell sorting).

| Antigen | Antigen Localization | Fluorescent dye | Antibody clone | Source |
| --- | --- | --- | --- | --- |
| CCR7 | Cell membrane | FITC | G043H7 | Biolegend |
| CD25 | Cell surface | PE | BC96 | Biolegend |
| CD3 | Cell surface | PE/Cyanine7 | UCHT1 | Biolegend |
| CD8 | Cell surface | PerCP-Cyanine5.5 | RPA-T8 | Biolegend |
| CD127 | Cell surface | APC | A019D5 | Biolegend |
| CD4 | Cell surface | APC/Fire750 | RPA-T4 | Biolegend |
| CD95 | Cell surface | Brilliant Violet 421 | DX2 | Biolegend |
| CD45RA | Cell surface | Brilliant Violet 510 | HI100 | Biolegend |

**Supplementary Table 4.**

|  | forward | Reverse |
| --- | --- | --- |
| *IKZF2* | 5′-GCTCCTCGCTGAAGATGGAG-3′ | 5′-TGCCTAACGTG TGTTTGTGC-3′ |
| *ACTB* | 5′-GATCATTGCTCCTCCTGAGC-3′ | 5′-CGTCATACTCCTG CTTGCTG-3′ |
| *RPL13A* | 5′-CGCAGATCACCCAGAAGATCG-3′ | 5′-TTCGTCGCATTTGTCCACCA-3′ |
| *TBP* | 5′-TGCACAGGAGCCAAGAGTGAA-3′ | 5′-CACATCACAGCTCCCCACCA-3′ |
